# The involvement of MsmK in pathogenesis of the *Streptococcus suis* serotype 2

**DOI:** 10.1002/mbo3.433

**Published:** 2017-01-19

**Authors:** Mei‐Fang Tan, Wan‐Quan Liu, Chun‐Yan Zhang, Ting Gao, Lin‐Lin Zheng, De‐Xin Qiu, Lu Li, Rui Zhou

**Affiliations:** ^1^ State Key Laboratory of Agricultural MicrobiologyCollege of Veterinary MedicineHuazhong Agricultural UniversityWuhanChina; ^2^ Institute of Animal Husbandry and Veterinary MedicineJiangxi Academy of Agricultural SciencesNanchangChina; ^3^ Institute of Animal Husbandry and Veterinary ScienceHubei Academy of Agricultural SciencesWuhanChina; ^4^ Cooperative Innovation Center of Sustainable Pig ProductionWuhanChina

**Keywords:** bloodstream, MsmK, pathogenesis, *Streptococcus suis*

## Abstract

*Streptococcus suis* serotype 2 (SS2) is an important swine and human pathogen that causes global economic and public health problems. Virulent *S. suis* strains successfully maintain high bacterial concentrations in host blood and rapidly adapt to challenging environments within hosts. Successful survival in hosts is a major factor influencing the pathogenesis of SS2. We have previously identified that SS2 colonization in mouse brain is possibly affected by the ATPase, MsmK of carbohydrate ATP‐binding cassette (ABC) transporters because of carbohydrate utilization. In this study, the chain length of the *msmK* deletion mutant was longer than that of the wild type, and the former was significantly more susceptible than the latter when theses strains were exposed to mouse blood both in vivo and in vitro. The hemolytic activity of the mutant strain was decreased. Although the adhesion of the mutant to HEp‐2 cell lines was enhanced, the deletion of *msmK* impaired the abilities of SS2 to resist phagocytosis and survive severe stress conditions. MsmK contributed to the survival and adaptation of SS2 in host bloodstream. Therefore, MsmK was identified as a multifunctional component that not only contributed to carbohydrate utilization but also participated in SS2 pathogenesis.

## 1 Introduction

*Streptococcus suis* serotype 2 (SS2) is a leading bacterial pathogen in the swine industry and cause of annual significant economic losses worldwide (Gottschalk, Xu, Calzas, & Segura, 2010). As the primary pathogenic agent of meningitis, septicemia and arthritis, SS2 has spread over 30 countries or regions and has been accounted for approximately 1,600 human cases (Feng et al., 2014; Goyette‐Desjardins, Auger, Xu, Segura, & Gottschalk, 2014). Insidiously colonizing healthy individuals, this pathogen must breach epithelial barriers, reach and survive in the bloodstream, and invade multiple organs. These phenomena result in systemic spread and stimulate exaggerated inflammation (Tang et al., 2006; Wertheim, Nghia, Taylor, & Schultsz, 2009).

SS2 naturally inhabits the upper respiratory tract, particularly tonsils and nasal cavities, and opportunistically translocates across the mucosal epithelium to reach the bloodstream (Gottschalk et al., 2010). Once reaching the bloodstream, SS2 is challenged by changing nutritional and immunological conditions, especially immunoglobulin‐induced opsonization and complement system activities (Doran et al., 2016). SS2 successfully survive in the host bloodstream to initiate inflammatory reactions that can lead to a toxic shock‐like syndrome, connective tissue invasion, and translocation into the central nervous system (Fittipaldi, Segura, Grenier, & Gottschalk, 2012). Therefore, survival in the blood is an important virulence trait of SS2 as a meningeal pathogen.

*S. suis* should adjust its metabolism to maximize nutrient availability in different host compartments (Willenborg et al., 2015). The mammalian brain contains plenty of glycogen as the sole carbohydrate reserve, which could sustain the growth of SS2 under infection conditions (Brown, 2004; Ferrando et al., 2014). By contrast, glucose concentrations ranging from 4.4 mmol/L to 6.6 mmol/L are present in the bloodstream, where SS2 undergoes metabolism favorable for its optimal growth in a glucose‐containing medium (Ferrando et al., 2014). *S. suis* can sense the nutrient supply and subsequently modulate the expression of its virulence factors. Capsular polysaccharide (CPS) expression increases when bacteria are grown in pig blood rather than in cerebrospinal fluid (Wu et al., 2014). CPS expression is also directly associated with carbohydrate metabolism in gram‐positive bacteria (Willenborg et al., 2011). Nineteen predicted or confirmed virulence factors, including suilysin, are expressed at high levels when SS2 is supplied with starch or pullulan instead of glucose as a sole carbon source (Ferrando et al., 2014). Thus, carbohydrate availability affects the pathogenetic performance of SS2.

Nearly 60 bacterial components are involved in the infection and pathogenicity of SS2 (Feng et al., 2014; Fittipaldi et al., 2012). A series of virulence factors plays determined or predicted roles in carbohydrate metabolism. ApuA as a bifunctional amylopullulanase of SS2 mediates adhesion to porcine epithelium and mucus (Ferrando, Fuentes, de Greeff, Smith, & Wells, 2010). CcpA as a catabolite control protein significantly affects the capsule synthesis and virulence properties of SS2 (Willenborg et al., 2011). The mutants of other carbohydrate metabolism‐related proteins, such as sucrose operon repressor (ScrR) (Wilson et al., 2007), sucrose‐6‐phosphate hydrolase (ScrB) (Wilson et al., 2007), sucrose phosphorylase (GtfA) (Wilson et al., 2007), glutamate dehydrogenase (Okwumabua, Persaud, & Reddy, 2001), and mannose‐specific transport PTS IID (ManN) (Wilson et al., 2007), have weakened virulence in animal infection models. However, most of these streptococcal carbohydrate‐active pathogenetic factors remain unexamined (Abbott et al., 2010). Further studies on metabolic systems should be conducted to enhance our understanding of the pathogenesis of SS2 infections.

We proposed that MsmK, an ATPase of ABC transporters that transport the degradation products of glycogen for SS2, possibly contributes to the colonization of SS2 in mouse brain because of glycogen utilization (Tan, Gao, Liu, Zhang, & Yang, 2015). We further identified the additional role played by MsmK in the bloodstream because different nutritional conditions exist in host blood and brain. Our results revealed that MsmK is involved in the resistance of SS2 to eradication in host blood and adaptation in host. Our study increased the number of carbohydrate metabolism components known to play specific roles in pathogenesis. Our study also provided further insights into the relationship between carbohydrate contents and SS2 pathogenesis.

## 2 Materials and Methods

### 2.1 Ethics statement

All mice used in this study were approved by Wuhan Institute of Biological Products. The license number was SYXK(E) 2010‐0029. The animal experiment protocol was approved by the Ethics Committee of Huazhong Agricultural University according to Hubei Province Laboratory Animal Management Regulations ‐ 2005.

### 2.2 Bacterial strains, culture media, and chemicals

SS2 SC‐19 strain was isolated from a sick pig during an epidemic outbreak in 2005 in Sichuan, China (Li, Liu, Chen, & Zhou, 2009). The *msmK* deletion mutant of SC‐19 (Δ*msmK*, with erythromycin resistance) and the complemented strain of Δ*msmK* (CΔ*msmK*, with erythromycin and spectinomycin resistance) were also used in this study (Tan et al., 2015). Under normal conditions, the SS2 strains were grown at 37°C in tryptic soy broth (TSB; Difco, France) or on tryptic soy agar (TSA; Difco) plates with 10% (v/v) newborn bovine serum (NBS; Sijiqing, Hangzhou, China). Erythromycin (90 μg/ml), spectinomycin (100 μg/ml), and streptomycin (20 μg/ml) were supplemented to promote bacterial selection, as appropriate. Chemicals were purchased from Biosharp (Hefei, China) unless otherwise specified.

### 2.3 Scanning electron microscopy

Scanning electron microscopy (SEM) assays were performed in accordance with previously described methods (Shi et al., 2014). SC‐19, Δ*msmK*, and CΔ*msmK* were grown at 600 nm optical density (OD_600_) of 0.8 (mid‐log phase) and spotted onto polylysine coverslips (WHB, Shanghai, China). The bacteria were fixed with 2.5% glutaraldehyde at 4°C overnight. The subsequent dehydration steps with ethanol were conducted as follows: 30% for 15 min, 50% for 15 min, 70% for 15 min, 90% for 15 min, 100% for 15 min, and 100% for 15 min. The dried samples were covered with a 10 nM thick gold layer and observed with a JSM‐6390LV SEM (NTC, Tokyo, Japan).

### 2.4 Transmission electron microscopy

Transmission electron microscopy (TEM) assays were performed in accordance with previously described methods (Gao et al., 2016). SC‐19, Δ*msmK*, and CΔ*msmK* were harvested at OD_600_ of 0.8 and fixed with 2.5% glutaraldehyde overnight. The samples were then treated with 2% osmium tetroxide for 2 hr and dehydrated in a serial dilution of ethanol. The dehydrated cells were embedded in epoxy resin and their morphological characteristics were observed using an H‐7650 TEM (Hitachi, Tokyo, Japan). Twenty bacterial cells from each strain were randomly chosen from the TEM micrographs to measure the thickness of capsule using Image J. The cells were then statistically analyzed with GraphPad Prism 6.

### 2.5 Bacterial survival in mouse blood after infection

Survival assay was performed in accordance with previously described methods (Marion, Aten, Woodiga, & King, 2011; Tan et al., 2015). Four‐ to six‐week‐old specific‐pathogen‐free (SPF) female Kun‐Ming mice were inoculated intraperitoneally with 3 × 10^7^ mid‐log‐phase cells at a 1:1 mixture of SC‐19 and Δ*msmK*. Five control mice were inoculated with normal saline. Five mice from each group were killed through carbon dioxide asphyxiation at 12 hr, 1 day, 3 days, or 5 days postinfection. Blood samples were collected, serially diluted, vortexed, and plated on TSA plates. TSA with streptomycin was prepared for the wild‐type (WT) SC‐19, whereas TSA with streptomycin and erythromycin was selective for Δ*msmK*. Colonies were counted and presented as colony‐forming unit (CFU)/ml. Data were drafted and analyzed with GraphPad Prism 6.

### 2.6 In vitro bacterial survival in the presence of mouse whole blood

Assays were performed in accordance with previously described methods (de Buhr, Neumann, Jerjomiceva, von Kockritz‐Blickwede, & Baums, 2014). SC‐19, Δ*msmK*, and CΔ*msmK* were cultured in the early stationary phase (OD_600_ of 1.2). The bacteria were suspended in normal saline at OD_600_ of 0.2. Subsequently, 1 ml of whole blood or 1 ml of TSB with 10% NBS was mixed with 100 μl of SS2 cells and incubated for 2 hr at 37°C. The incubated mixtures were harvested at 0 and 2 hr, serially diluted, vortexed, and plated onto TSA plates to determine the bacterial survival factors. Survival factors were calculated as the ratio of the data at 2 hr to the data at 0 hr. *S. suis* strains grown in broth were regarded as positive control. The data at 0 hr were considered as 1. The assays were performed in triplicate and repeated thrice.

### 2.7 Hemolytic activity detection

Hemolytic activity (HA) was detected in accordance with previously described methods (Ferrando et al., 2014). The supernatant was collected from 1 ml of cultures in the early stationary phase through centrifugation at 12,000*g* for 1 min. Each 100 μl of the supernatant was incubated for 2 hr at 37°C with 100 μl of 2% washed sheep erythrocytes. Unlysed red blood cells were allowed to pellet through centrifugation. Afterward, 100 μl of the supernatant was transferred to a sterile new microplate. Absorption was determined at 550 nm in a microplate reader (Biotek, Vermont, USA). Sterile culture media were used as negative controls. The experiments were conducted in triplicate to detect HA and repeated at least thrice.

### 2.8 Adherence and invasion assays

Adherence and invasion assays were conducted in accordance with previously described methods (Li, Wan, Tao, Chen, & Zhou, 2013). HEp‐2 cells (1.6 × 10^5^ cells per well) were infected with log‐phase SS2 strains (OD_600_ of 0.8) to reach a multiplicity of infection (MOI) of 100:1 (bacteria:cells) and incubated at 37°C for 2 hr. HEp‐2 cells were then lysed in 1 ml of sterile distilled water to count the adherent and intracellular bacteria. The appropriate diluted lysates were vortexed and plated on TSA plates. HEp‐2 cells were also incubated with ampicillin (100 μg/ml) for 2 hr before lysis. Plating was performed to count the intracellular bacteria alone. The experiments were performed in triplicate wells. All experiments were performed thrice.

### 2.9 Phagocytosis assay

Phagocytosis assay was performed in accordance with previously described methods (Li et al., 2013). RAW264.7 cells were infected with log‐phase SS2 strains (OD_600_ of 0.8) to reach a MOI of 10:1. Penicillin (100 μg/ml) was added for 2 hr of incubation to kill the extracellular bacteria before lysis and plating were performed. Viable intracellular bacteria were determined by plating serial dilutions on TSA plates. The experiments were performed in triplicate wells and repeated thrice.

### 2.10 Osmotic stress assay

The adaptability of the SS2 strains to osmotic stress was evaluated by monitoring bacterial growth in TSB containing 10% NBS with 0.4 mol/L NaCl in accordance with previously described methods (Zhu et al., 2014). The overnight cultures of SC‐19, Δ*msmK*, and CΔ*msmK* were diluted in a fresh medium with or without NaCl to obtain OD_600_ of 0.2. The samples were incubated at 37°C for 8 hr. Bacterial growth was monitored at 1 hr interval by determining OD_600_. The assays were performed in triplicate and repeated thrice.

### 2.11 Oxidative stress assay

The SS2 strains were challenged with H_2_O_2_ to evaluate oxidative stress tolerance in accordance with previously described methods (Zhu et al., 2014). The sensitivity to H_2_O_2_ of bacteria was examined by exposing culture aliquots to 0, 20, and 30 mmol/L H_2_O_2_ for 20 min at 37°C. The results were presented as the percentage of survival. The assays were performed in triplicate and repeated thrice.

### 2.12 RNA isolation, reverse transcription, and quantitative real‐time PCR (qRT‐PCR)

SC‐19 and Δ*msmK* were grown in an early stationary phase in a TSB medium to detect the expression levels of *sly*. SC‐19 and Δ*msmK* were grown in an early stationary phase in a TSB medium containing 10% NBS to analyze adherence. SC‐19 and Δ*msmK* were grown for 4.5 hr in a TSB medium containing 10% NBS with/without 0.4 mol/L NaCl for osmotic stress analysis. Total RNA was isolated using an SV total RNA isolation system (Promega, Shanghai, China) according to the manufacturer's instructions. cDNA was synthesized using reverse transcriptase mixture (Vazyme, Nanjing, China) according to the manufacturer's instructions. Parallel samples were processed without the addition of reverse transcriptase as a negative control.

In qRT‐PCR, primers (Table 1) were designed with Oligo version 6 (MedProbe, Oslo, Norway). The reactions were performed in an ABI 7500 system (Applied Biosystems, California, USA) under the following parameters: 95°C for 10 min, followed by 40 cycles of 95°C for 15 s, 56°C for 30 s, and 72°C 35 s. Melting curve analysis was then performed. The 16S rRNA gene was chosen as the internal control. The reactions were performed with three independent biological repeats and reference dye normalization. The relative expression level was measured with 2^−ΔΔCt^ method (Livak & Schmittgen, 2001).

**Table 1:** Primers used for qRT‐PCR in this study

Gene name	Locus tag	Forward primer sequence (5′–3′)	Reverse primer sequence (5′–3′)	Length (bp)	Reference or source
*sly*	SSUSC84_1264	GGAGATCTGACGCTTAGTT	CATATTGAAGCTCTGCTTGG	172	(Li et al., 2013)
*ccpA*	SSUSC84_1235	CTCACGTACACCGATTGTT	CTCACGTACACCGATTGTT	205	This work
*sadP*	SSUSC84_0242	GTAGAGAAGCAACAGTCGCC	GTTACTTTCCACTGAATCTG	137	This work
*gapdH*	SSUSC84_0136	AGAAGTAAACGCTGCTAT	CAAACAATGAACCGAAT	107	(Li et al., 2013)
*fbpS*	SSUSC84_1341	GGTGGCCCAGCAGGCCAATG	CCGCCAATCCCTGCTCCTGC	95	(Li et al., 2013)
*sao*	SSUSC84_1234	GATGAGCTTGGTGGTATTG	GATGAGCTTGGTGGTATTG	148	This work
*apuA*	SSUSC84_1871	GATGACTACGGCCATTACG	GATGACTACGGCCATTACG	151	This work
*dltA*	SSUSC84_0569	CAGTGCTTGGTTTGTCTGA	CAGTGCTTGGTTTGTCTGA	191	This work
*hylA*	SSUSC84_1087	GCTCTGAGGTTAGTCCTG	GCTCTGAGGTTAGTCCTG	124	This work
*grpE*	SSUSC84_0268	CCATTCAGCGTTACCGTTC	GACATCTGTTGCGACTTCTT	182	This work
*perR*	SSUSC84_0278	TTGAACACGTCATCCAACAT	GTAGTTAGGTATTAGATCTTG	137	(Zhang et al., 2012)
*gor*	SSUSC84_0448	GTTCACGCGCATCCTACG	TACCAGGAATAGCAGGGAC	171	This work
*nox*	SSUSC84_0648	GTTCACAACCTATCTTACCA	GTAACCAGCACCAACAACT	176	This work
*mscL*	SSUSC84_1286	CCTTGCTGTAGCAGTGGTA	ACTACCGTACTTCACACCA	157	This work
*sodA*	SSUSC84_1386	GAAGCCTTCACTGCAGCA	CCTTGCATGATTGGTGTGT	122	(Zhang et al., 2012)
*opuAa*	SSUSC84_1444	CCTGACGAGGATGAGAGA	GGTTCGTCCATGAGTAACA	156	This work
16S rRNA	SSUSC84_r0001	GTAGTCCACGCCGTAAACG	TAAACCACATGCTCCACCGC	160	(Li et al., 2009)

### 2.13 Biofilm formation assay

Biofilm formation assay was conducted in accordance with previously described methods (Grenier, Grignon, & Gottschalk, 2009). The overnight cultures were diluted at OD_600_ of 0.1 and 200 μl of each diluted culture was added to 96‐well microplates. The bacteria were incubated for 12 hr and 24 hr at 37°C. Afterward, the medium and free‐floating bacteria were removed and stained with 100 μl of 0.04% crystal violet for 10 min. The wells were washed to remove the unbound crystal violet dye and dried for 2 hr at 37°C. After 100 μl of 95% ethanol was added to each well, the plates were shaken for 10 min. Absorbance was recorded at 550 nm. The wells with a sterile medium were used as negative controls. The experiments were run in triplicate and repeated at least thrice.

### 2.14 Murine infection experiments

Fifty 4‐ to 6‐week‐old sPF female Kun‐Ming mice were randomly classified into five groups with 10 mice per group. The log phase cultures of SC‐19 and Δ*msmK* were diluted in normal saline to achieve the following concentrations: 1.0 × 10^9^ CFU/0.5 ml and 3.0 × 10^9^ CFU/0.5 ml. Afterward, 0.5 ml of inoculum was intraperitoneally introduced. Ten control mice were treated with normal saline. The infected mice were observed for 1 week and deaths were recorded.

### 2.15 Statistical analysis

Statistical analyses were performed via unpaired Student's *t*‐tests in GraphPad Prism 6. All data are expressed as mean ± SEM. Differences were considered significant on the basis of the calculated *p* value (ns, *p *> .05; _*_, *p* ≤ .05; _**_, *p* ≤ .01; _***_, *p* ≤ .001).

## 3 Results

### 3.1 Microbiological characterization of the mutant

**Figure 1:**
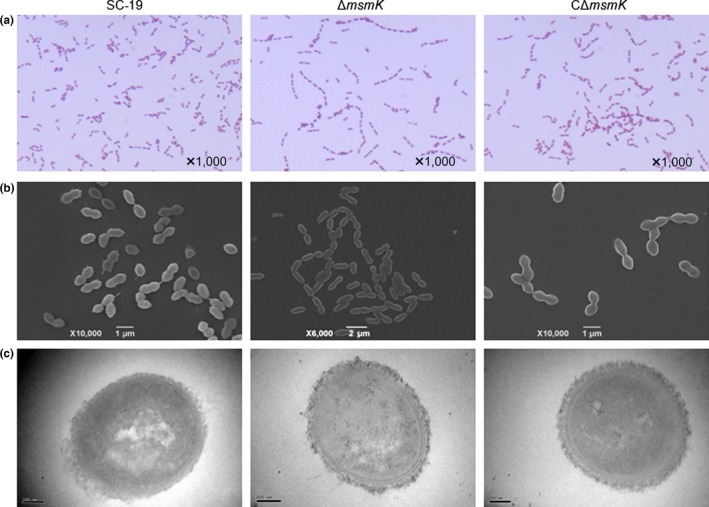
Characterization of SC‐19, Δ*msmK*, and CΔ*msmK*. (a) Gram‐stained images of SS2 strains under light microscopy. (b) Scanning electron micrographs of SS2 strains. (c) Transmission electron micrographs of SS2 strains. The bar means 100 nm

Morphological examination by Gram staining showed that MsmK mutation lengthened the bacterial chain (Figure 1a). The same phenotype was observed in subsequent SEM‐based assays (Figure 1b). The chain length of SS2 was partially restored after complementation. Furthermore, the thickness of the capsules of SC‐19 (47.2 ± 5 nm) and Δ*msmK* (50.6 ± 3 nm) was similar (Figure 1c).

### 3.2 MsmK contributes to SS2 survival in mouse blood both in vivo and in vitro

**Figure 2:**
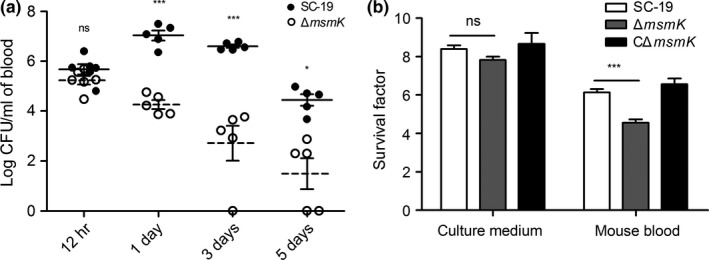
MsmK contributes to SS2 survival in mouse blood in vivo and in vitro. (a) In vivo survival assays. At each defined timepoint, blood samples of five mice were collected and viable bacteria were counted. SC‐19 and Δ*msmK* were distinguished by adding erythromycin to TSA plates. Solid lines correspond to the means of data related to SC‐19. Dotted lines correspond to the means of data related to Δ*msmK*. (b) Survival factors of SC‐19, Δ*msmK*, and CΔ*msmK* in culture medium and mouse whole blood. Colony‐forming unit (CFU) was determined at 0 and 2 hr of incubation on a rotator at 37°C. CFU at 0 hr was designated as 1. Survival factor was calculated as the ratio of the data at 2 hr to the data at 0 hr. Strains grown in culture medium were regarded as positive control. Data are presented as means ± SEM

Previous studies proposed that the mutation of *msmK* affects SS2 survival in infected mouse brains mainly because of the mutant loss of the ability to utilize glycogen in vivo (Tan et al., 2015). Nutritional and immunological conditions are different between the blood and the brain. The finding indicated that different survival performances of SS2 can be expected in the bloodstream. In our study, in vivo bacterial survival assays in the blood were conducted. In Figure 2a, Δ*msmK* manifested significant defects in the blood survival after 24 hr of infection. The maximum growth of Δ*msmK* was obtained at 12 hr after infection. By contrast, the peak timepoint of SC‐19 was 24 hr. To confirm the susceptibility of the mutant to eradication in the blood, we carried out in vitro assays. Compared with the survival factor of SC‐19, the survival factor of Δ*msmK* in mouse whole blood decreased significantly (Figure 2b). The results suggested that Δ*msmK* was significantly more susceptible than SC‐19 when they were exposed to mouse blood. Therefore, MsmK contributes to SS2 survival in mouse blood.

### 3.3 Reduced HA of the mutant

**Figure 3:**
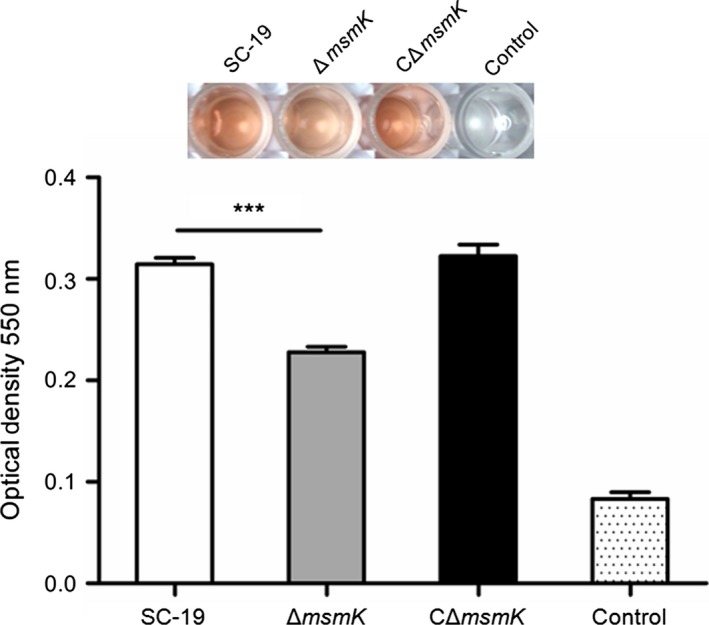
Hemolysis assay of SS2 strains. The hemolysis production was quantified by analyzing the supernatants collected from SS2 strains grown in culture medium. Data points are the mean ± SEM from three independent experiments performed in quintuplicate

We determined the erythrocyte HA of the supernatants of SS2 strains. In Figure 3, the HA of Δ*msmK* was significantly lower than that of SC‐19 and CΔ*msmK*. The expression level of *sly* of Δ*msmK* was also lower (−3.46‐fold change) than that of SC‐19. This finding indicated that the absence of MsmK could influence the HA of SS2.

### 3.4 *msmK* absence promotes adhesion in vitro

**Figure 4:**
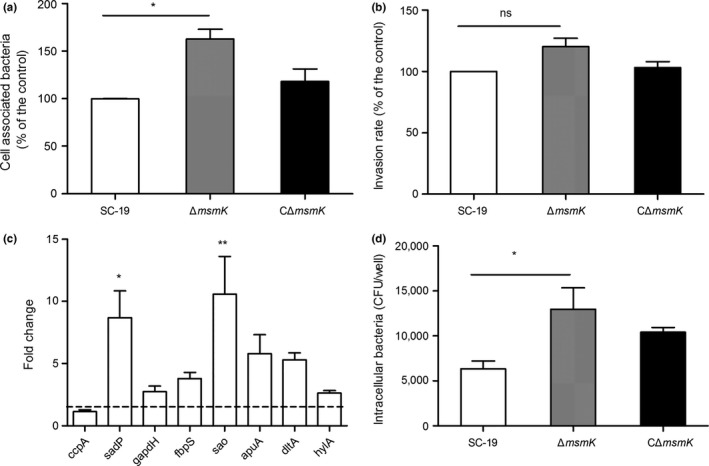
*msmK* absence promotes adhesion to Hep‐2 cells and susceptibility to macrophages. (a) Cell‐associated bacteria recovered after incubation with HEp‐2 cells. The number of the recovered SC‐19 serves as the control, which was regarded as 100% cell association. Up to 100% cell association represents 83,600 colony‐forming unit (CFU)/well. Data were expressed as mean ± SEM. *, p ≤ 0.05. (b) Pathogen invasion of HEp‐2 cells. Extracellular bacteria were eradicated through antibiotic treatment. The number of the recovered intracellular SC‐19 serves as the control, which was regarded as 100% cell invasion. Up to 100% cell invasion represents the entry of 3,060 CFU/well. Data are expressed as mean ± SEM. ns, p > 0.05. (c) Gene expression analysis of adherence‐related genes of Δ*msmK* compared with SC‐19. The dotted line means +2‐fold change. The height of the bars indicates the mean values for the relative expression data ± SEM. *, p ≤ 0.05; **, p ≤ 0.01. (d) Phagocytosis of SS2 strains by RAW264.7 cells. Phagocytosed bacteria were recovered after antibiotic treatment. Data are presented as mean ± SEM. *, p ≤ 0.05

We investigated whether Δ*msmK* changes its abilities to adhere and invade host cells. The adherence efficiencies of SC‐19 and its derivatives to the HEp‐2 cells were calculated to determine whether the absence of *msmK* affects the cellular adhesion of SS2. The binding rate of Δ*msmK* to the HEp‐2 cells was 1.5‐fold higher than that of SC‐19 (Figure 4a). By contrast, the *msmK* mutant did not significantly differ from the WT strain in the invasion assay (Figure 4b).

Transcriptional analysis revealed that *sadP*, an adhesion that mediates the binding of SS2 to galactosyl‐α1‐4‐galactose‐containing host receptors (Kouki et al., 2011), is upregulated to a higher extent in Δ*msmK* than that in SC‐19 (Figure 4c). The expression levels of other known adhesion factors, such as *gapdH*,* fbpS*,* aupA*, and *dltA*, also increased (Figure 4c). These data suggested that the mutation of *msmK* promotes adhesion in vitro.

### 3.5 Susceptibility to macrophages

Δ*msmK* was more likely phagocytosed by phagocytic cells than the WT (Figure 4d). This result indicated that *msmK* inactivation significantly decreased the resistance of SS2 to phagocytosis.

### 3.6 MsmK deficiency affects the osmotic and oxidative tolerance of SS2

**Figure 5:**
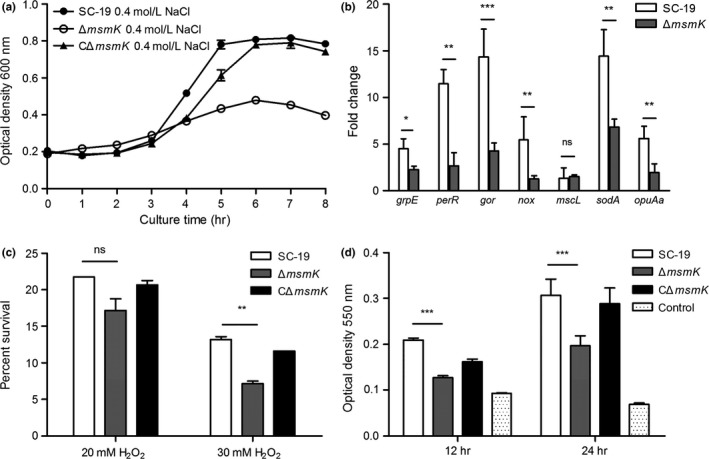
In vitro stress assays. (a) Osmotic stress assay. Growth curves were monitored at an initial OD_600_ of 0.2. SS2 strains were grown at 400 mmol/L NaCl. Data points are the mean ± SEM from three independent experiments performed in triplicate. (b) Gene expression analysis of osmosis‐related genes of SC‐19 and Δ*msmK* in the presence of 400 mmol/L NaCl compared with the absence of NaCl. The results represent the mean ± SEM. ns, p > 0.05; *, p ≤ 0.05; **, p ≤ 0.01; ***, p ≤ 0.001. (c) Oxidative stress assay. Bacteria were incubated at 37°C for 20 min with or without 20 mmol/L or 30 mmol/L H_2_O_2_. Data are presented as mean survival rate ± SEM. ns, p > 0.05; **, p ≤ 0.01. (d) Biofilm formation by SS2 strains. Strains were incubated in 96‐well microplates for 12 and 24 hr at 37°C. The wells with a sterile medium served as negative controls. Data are expressed as mean ± SEM. ***, p ≤ 0.001

We determined the osmotic and oxidative tolerance of SC‐19, Δ*msmK* and CΔ*msmK* under stress conditions in vitro. Compared with that of SC‐19 and CΔ*msmK*, the growth of Δ*msmK* in 0.4 mol/L NaCl was remarkably inhibited (Figure 5a). A series of genes related to osmotic defence was selected for qRT‐PCR assays (Table 1). In Figure 5b, the gene expression levels were higher in both strains grown in 0.4 mol/L NaCl than those of the strains grown without NaCl. The fold changes of Δ*msmK* were significantly less than those of SC‐19. As a result, the tolerance of Δ*msmK* to osmotic stress decreased. These data revealed that MsmK contributed to the resistance of SS2 to osmotic stress.

In contrast to the high sensitivity to NaCl, the sensitivity to 20 mmol/L H_2_O_2_ of the mutant was similar to that of the WT strain (Figure 5c). The survival rate of Δ*msmK* decreased significantly at 30 mmol/L H_2_O_2_ (Figure 5c). This result revealed that MsmK contributed to the resistance of SS2 to oxidative stress to some extent.

### 3.7 MsmK deficiency influences biofilm formation

The formation of biofilms by microorganisms is a mechanism that allows them to become persistent colonizers, resist clearance by the host immune system, enhance resistance to antibiotics, and exchange genetic materials (Grenier et al., 2009). In this study, the ability of Δ*msmK* to form biofilms was weaker than that of SC‐19 after these strains were incubated for 12 or 24 hr (Figure 5d). Therefore, *msmK* also plays a role in the biofilm formation of SS2.

### 3.8 Absence of MsmK impairs SS2 infection in mice

**Figure 6:**
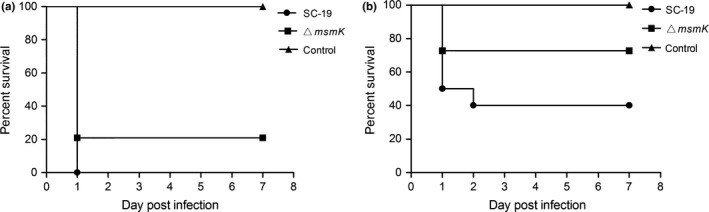
Survival rates of the mice challenged with SC‐19 or Δ*msmK*. Each mouse was intraperitoneally inoculated with 3.0 × 10^9^ CFU (a) and 1.0 × 10^9^ CFU (b) of SC‐19 and Δ*msmK*, respectively. The mice inoculated with normal saline served as controls. Ten mice were used in each group. CFU, colony‐forming unit

The half lethal dose (LD_50_) of SC‐19 in mice is 1.5 × 10^9^ CFU (Li, Hu, Liu, Chen, & Zhou, 2011). Mice were intraperitoneally inoculated in parallel with a low dose (0.7 ×  LD_50_) of SC‐19/Δ*msmK* and a high dose (2 × LD_50_) of SC‐19/Δ*msmK* to evaluate the effect of MsmK on SS2 infection. In Figure 6, the survival rate of the mutant was higher than that of the WT at the same dosage. This result indicated that the mutant became less virulent than SC‐19.

## 4 Discussion

Bacterial carbohydrate metabolism components have been associated with environmental survival, colonization, host–pathogen interaction, and virulence, such as those observed in *S. suis* (Ferrando et al., 2010, 2014), *Streptococcus pneumoniae* (Marion, Burnaugh, Woodiga, & King, 2011; Marion, Aten, et al., 2011), *Streptococcus mutans* (Klein et al., 2010), and group A streptococcus (Shelburne et al., 2009). Among the known ones are pullulanases and the lipid‐anchored solute‐binding proteins (Abbott et al., 2010). In this study, MsmK, an ATPase of carbohydrate ABC transporters, is a potential pathogenic factor involved in blood killing and host adaptation of SS2.

Figure 2a shows that the survival of the *msmK* mutant in host blood remarkably decreased 24 hr after infection compared with SC‐19. The survival of the *msmK* mutant in host blood was also significantly lower than that of SC‐19 in the following days. Figure 2b shows that the survival of the mutant in whole blood decreased significantly in vitro. These results indicated that MsmK was involved in the survival of this pathogen in host blood. CPS is essential for SS2 survival in blood because of its strong anti‐phagocytic properties (Doran et al., 2016). We observed the capsules of SC‐19 and Δ*msmK* with TEM and found that the thickness of capsules of the WT and the mutant were similar (Figure 1c). Previous studies demonstrated that an increase in the size of pathogens promotes complement‐dependent eradication by either natural changes in cellular morphology or antibody‐mediated agglutination (Dalia & Weiser, 2011). These observations provide insights into the effects of cell size and morphology on the pathogenesis of pathogenic microbes. Gram staining and SEM revealed that the bacterial chain length of the *msmK* mutant significantly increased (Figure 1a and b). This result may be one of the important reasons that decreased the resistance of Δ*msmK* to blood killing and phagocytosis (Figures 2 and 4d), because pathogens are sensitized by an increase in bacterial chain length to complement the deposition and subsequent uptake by host neutrophils (Dalia & Weiser, 2011). The failure of maintenance in the bloodstream could be an important reason for the reduced lethality of Δ*msmK* (Figure 6).

Chain length is also an important factor in adherence to epithelial cells in vitro and colonization in vivo. Long chain enhances the ability of *S. pneumoniae* to bind to the epithelial surface and promotes colonization of the host upper respiratory tract (Rodriguez, Dalia, & Weiser, 2012). The chains formed by the *msmK* mutant were longer than those produced by SC‐19. As such, the mutant likely showed an increased association with HEp‐2 cells in vitro (Figure 4a). The expression levels of several genes involved in adherence were also higher in the mutant than in SC‐19 (Figure 4c), which also contributed to the ability of Δ*msmK* to bind to epithelial cells.

With the increase in association, the mutant exhibited no evident changes in its invasion of epithelial cells (Figure 4b). The HA of the mutant was reduced significantly (Figure 3), and this result may be responsible for pathogen invasion. Suilysin plays an important role in damaging host epithelial, endothelial, and immune cells. This condition suggested that suilysin could promote tissue invasion and inhibition or killing of leukocytes in vivo (Benga, Fulde, Neis, Goethe, & Valentin‐Weigand, 2008; He et al., 2014; Lecours et al., 2011). Thus, the decreased expression of suilysin may also result in weak resistance to blood killing.

During disease development, SS2 likely encounters adverse environmental conditions, such as increased osmolality and oxidative pressure. We investigated the characteristics of Δ*msmK* under stress conditions. Compared with that of SC‐19 and CΔ*msmK*, the growth of Δ*msmK* in 0.4 mol/L NaCl was remarkably inhibited (Figure 5a). qRT‐PCR further revealed that the expression levels of the related genes were upregulated more remarkably in SC‐19 than in Δ*msmK* after the strains were exposed to NaCl (Figure 5b). The downregulation of stress response genes may be attributed to the decreased stress tolerance of Δ*msmK*. The resistance to high H_2_O_2_ levels of the mutant was also weak (Figure 5c). The decreased tolerance of Δ*msmK* to environmental stresses may be an important factor causing the defective survival of the mutant in the bloodstream in vivo (Figure 1a) and brain (Tan et al., 2015) in late infection stages, because mutant bacteria may be less likely to adapt to edematous and pathological environments postinfection.

*S. suis* is considered a major swine pathogen increasingly isolated from a wide range of mammalian species, including humans, and birds. *S. suis* is also a normal inhabitant of the intestines of various ruminants (Gottschalk et al., 2010). These findings suggested that the infection exhibits complex epidemiological patterns and diverse pathogenesis in several species. The animal model used in this study revealed that MsmK promoted pathogenic infection in mice. Adhesion experiments also demonstrated the possibility that MsmK is involved in pathogenic infections in humans. The pathogenicity of strains infecting pigs should be further investigated because awareness regarding the role of carbohydrate components in *S. suis* pathogenesis may provide insights into novel strategies to eradicate this pathogen.

In summary, MsmK contributed to the resistance to blood killing, phagocytosis, and severe stress, enhanced HA, and promoted the ability of SS2 to infect its hosts (Figures 2, 3, 4, 5, 6). This study not only explained the increased sensitivity of Δ*msmK* to host blood but also revealed a link between MsmK and SS2 pathogenesis. The interactive role of MsmK played in the carbohydrate metabolism and pathogenesis of SS2 should be further examined. A series of proteins involved in carbohydrate metabolism directly or indirectly contributes to the pathogenesis/virulence of SS2 (Feng et al., 2014; Ferrando et al., 2010; Willenborg, de Greeff, Jarek, Valentin‐Weigand, & Goethe, 2014). Our findings may enhance our understanding of the complicated interplay between carbon metabolic components and complex pathogenic processes of SS2.

## Conflict of Interest

Authors declare no conflict of interest.
